# Crystal structure and Hirshfeld surface analysis of a copper(II) complex containing 2-nitro­benzoate and tetra­methyl­ethylenedi­amine ligands

**DOI:** 10.1107/S2056989021002802

**Published:** 2021-03-19

**Authors:** Sevgi Kansiz, Adnan M. Qadir, Necmi Dege, Li Yongxin, Eiad Saif

**Affiliations:** a Department of Fundamental Sciences, Faculty of Engineering, Samsun University, 55420, Samsun, Turkey; b Department of Chemistry, College of Science, Salahaddin University, Erbil, 44001, Iraq; c Department of Physics, Faculty of Arts and Sciences, Ondokuz Mayıs University, 55139, Samsun, Turkey; d Division of Chemistry and Biological Chemistry, Nanyang Technological University, 637371, Singapore; e Department of Computer and Electronic Engineering Technology, Sana’a Community College, Sana’a, Yemen; f Department of Electrical and Electronic Engineering, Faculty of Engineering, Ondokuz Mayıs University, 55139, Samsun, Turkey

**Keywords:** crystal structure, copper(II), tetra­methyl­ethylenedi­amine, 2-nitro­benzoate, Hirshfeld surface

## Abstract

The reaction of copper(II) sulfatepentahydrate with 2-nitro­benzoic acid and *N*,*N*,*N*′,*N*′-tetra­methyl­ethylenedi­amine (TMEDA) in basic solution produces the complex bis­(2-nitro­benzoato-κ*O*)(*N*,*N*,*N*′,*N*′-tetra­methyl­ethylenedi­amine-κ^2^ *N*,*N*′)copper(II), [Cu(C_7_H_4_NO_4_)_2_(C_6_H_16_N_2_)] or [Cu(2-nitro­benzoate)_2_(tmeda)]. Each carboxyl­ate group of the 2-nitro­benzoate ligand is coordinated by Cu^II^ atom in a monodentate fashion and two TMEDA ligand nitro­gen atoms are coordinate by the metal center, giving rise to a distorted square-planar coordination environment. In the crystal, metal complexes are linked by centrosymmetric C—H⋯O hydrogen bonds, forming ribbons *via* a *R*_2_^2^(10) ring motif. These ribbons are linked by further C—H⋯O hydrogen bonds, leading to two-dimensional hydrogen-bonded arrays parallel to the *bc* plane. Weak π–π stacking inter­actions provide additional stabilization of the crystal structure. Hirshfeld surface analysis, *d_norm_* and two-dimensional fingerprint plots were examined to verify the contributions of the different inter­molecular contacts within the supra­molecular structure. The major inter­actions of the complex are O⋯H/H⋯O (44.9%), H⋯H (34%) and C⋯H (14.5%).

## Abstract 2

In the title complex, [Cu(2-nitro­benzoate)_2_(tmeda)], the central metal atom has distorted square-planar geometry with one oxygen atom each from two 2-nitro­benzoate ligands and two TMEDA ligand nitro­gen atoms.

## Chemical context   

Copper(II) carboxyl­ate complexes continue to be of considerable inter­est on account of their biological properties such as anti­bacterial (Melník *et al.*, 1982 ▸), anti­fungal (Kozlevčar *et al.*, 1999 ▸), cytotoxic and anti­viral activities (Ranford *et al.*, 1993 ▸). Carboxyl­ate ligands are versatile and can coordinate to metal centers in different modes such as monodentate, bidentate and bridging fashions. The bidentate coordination can be either symmetrical bidentate chelating, having the same C—O bond lengths, or asymmetrical bidentate chelating, having different C—O bond lengths. Carboxyl­ate ligands have been used to generate units for developing supra­molecular architectures. Copper is one of essential metals for human life. In the human body, various enzymes are copper-dependent such as Cytochrome c oxidase, superoxide dismutase, ferroxidases, mono­amine oxidase, and dopamine β-monoxygenase (Brewer, 2009 ▸; Balamurugan & Schaffner, 2006 ▸). In this work, a new copper(II) complex involving 2-nitro­benzoic acid and *N*,*N*,*N*′,*N*′-tetra­methyl­ethylenedi­amine was synthesized, characterized by single crystal X-ray and studied by Hirshfeld surface analysis.
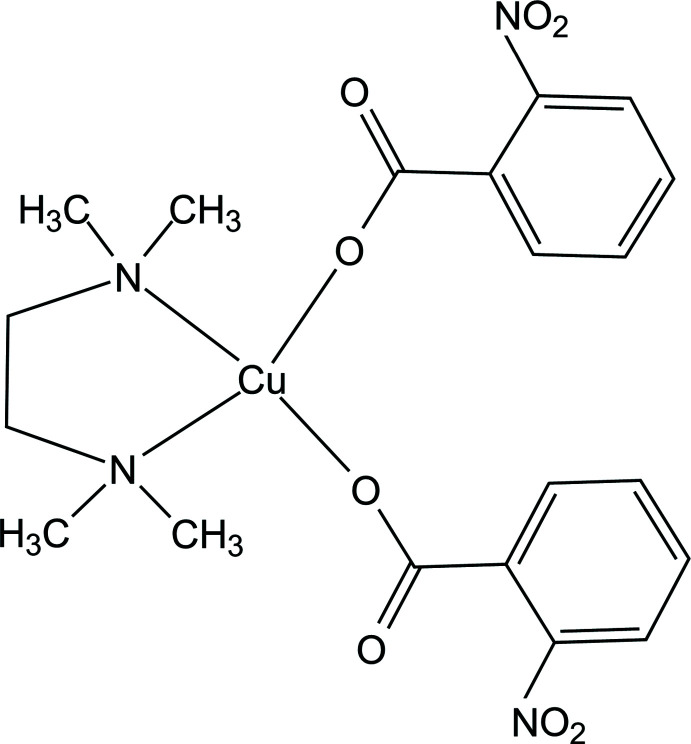


## Structural commentary   

**Figure 1:**
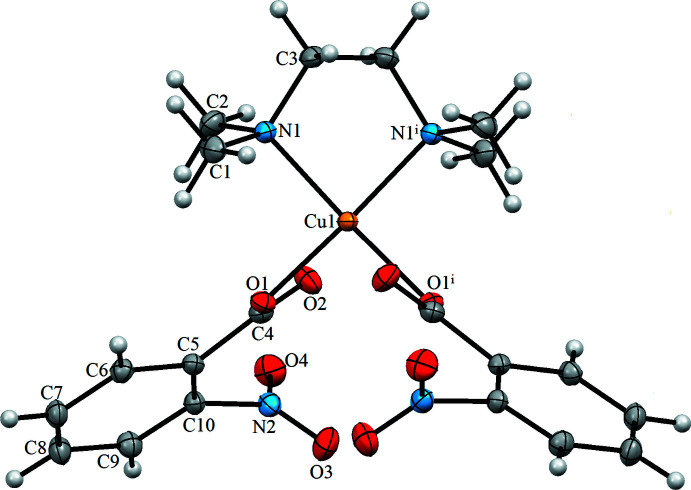
The mol­ecular structure of [Cu(2-nitro­benzoate)_2_(tmeda)], with the atom labeling. Displacement ellipsoids are drawn at the 30% probability level. Symmetry code: (i) −*x* + 1, *y*, −*z* + 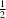.

Copper(II) acetate reacts with 2-nitro­benzoic acid and *N*,*N*,*N*′,*N*′-tetra­methyl­ethylenedi­amine (TMEDA) to give the mono-nuclear copper(II) complex (I). The asymmetric unit of the title compound contains one half of the metal complex, the central metal being located on the special position 4*e* (1/2, *y*, 1/4). The Cu^II^ atom has a distorted square-planar geometry with one oxygen atom each from two nitro­benzoic acid ligands and two TMEDA ligand nitro­gen atoms (Figs. 1 ▸ and 2 ▸). The two nitro groups of the rings are oriented *trans* to each other, being symmetry-related to each other through a twofold axis. The structure of the complex is shown in Fig. 1 ▸. The Cu1—N1 and Cu1—O1 bond distances are 2.0269 (13) and 1.9589 (11) Å, respectively. The structural parameters of the TMEDA ligand, *i.e*. Cu—N bond lengths, are in agreement with a work reported by Gumienna-Kontecka *et al.* (2013 ▸). The C4—O1 and C4—O2 distances in the carboxyl group are 1.2772 (19) and 1.2388 (18) Å, respectively. Selected bond lengths are given in Table 1 ▸.

## Supra­molecular features   

**Figure 2:**
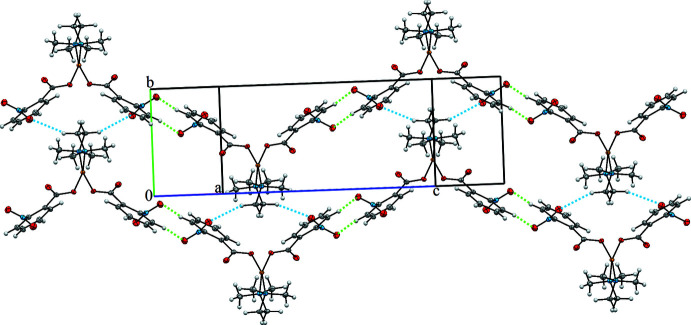
View of the two-dimensional hydrogen-bonded network in the structure of [Cu(2-nitro­benzoate)_2_(tmeda) showing C9—H9⋯O4 hydrogen bonds [described by an 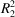
(10) ring motif] as green dashed lines and C3—H3*A*⋯O3 hydrogen bonds as blue dashed lines.

The crystal packing of the title complex (Fig. 2 ▸) features inter­molecular hydrogen bonds (C3—H3*A*⋯O3^i^ and C9—H9⋯O4^ii^; symmetry codes as in Table 2 ▸). The metal complexes are self-assembled by centrosymmetric C9—H9⋯O4 hydrogen bonds along the *c*–axis direction, forming supra­molecular ribbons linked *via R*_2_^2^(10) ring motifs. Adjacent ribbons are connected by C3—H3*A*⋯O3 hydrogen bonds; these inter­actions lead to the formation of layers lying parallel to the *bc* plane. The three-dimensional network is stabilized by π–π stacking inter­actions with a centroid-to-centroid distance *Cg*1⋯*Cg*1^iii^ of 3.741 (2) Å, where *Cg*1 is the centroid of the C5–C10 ring [symmetry code: (iii) −*x* + 1, −*y* + 1, −*z* + 1].

## Database survey   

A search of the Cambridge Structural Database (CSD, version 5.41, update of November 2019; Groom *et al.*, 2016 ▸) for the title complex revealed four hits: *catena*-[(μ_2_-terephthalato-*O*,*O*′,*O*′′,*O*′′′)(μ_2_-terephthalato-*O*,*O*′′)bis­[*N*-(2-amino­eth­yl)-3-amino-1-propanol]dicopper(II)] (FEMBEF; Mukherjee *et al.*, 2004 ▸), bis­[(μ_2_-biphenyl-2,2′-di­carboxyl­ato-*O* ^2^,*O* ^2′^)[*N*-(pyr­id;in-2-yl-*N*)pyridin-2-amine-*N* ^1^]]dicopper(II) tetra­hydrate (GUCXOS; Kumagai *et al.*, 2009 ▸), bis­[(μ_2_-biphenyl-2,2′-di­carboxyl­ato-*O* ^2^,*O* ^2′^)[*N*-(pyridin-2-yl-*N*)pyridin-2-am­ine-*N* ^1^]]dicopper(II) biphenyl-2,2′-di­carb­oxy­lic acid solvate monohydrate (GUCXUY; Kumagai *et al.*, 2009 ▸) and bis­(2-nitro­benzoato)bis­(3,5-dimethyl-1*H*-pyrazole-*N* ^2^)copper(II) (MIJFUH; Karmakar *et al.*, 2007 ▸). The Cu—N and Cu—O bond lengths range from 1.973 to 2.022 Å and 1.955 to 1.987 Å, respectively. The Cu—N and Cu—O bond lengths in the title complex [2.0269 (13) and 1.9589 (11) Å, respectively] fall within these limits.

## Hirshfeld surface analysis   

**Figure 3:**
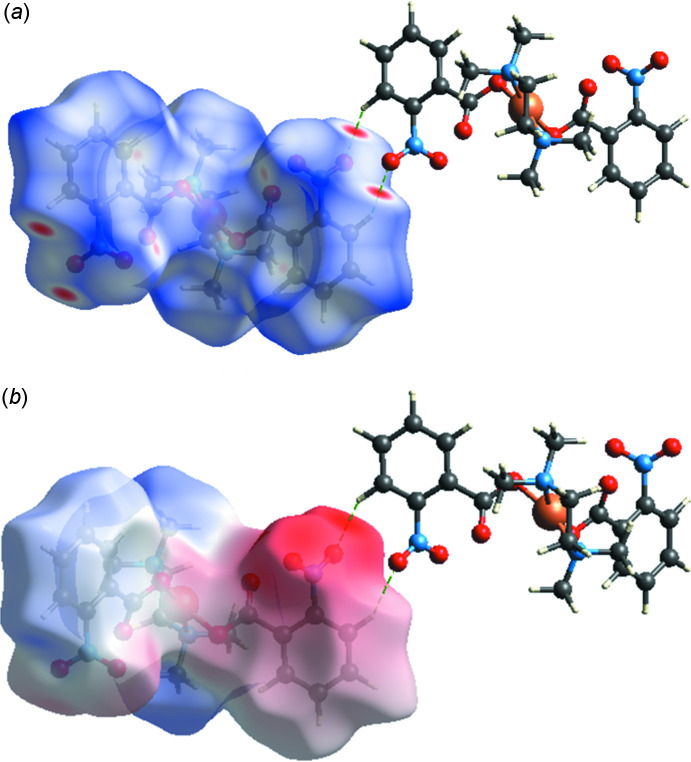
Hirshfeld surface of [Cu(2-nitro­benzoate)_2_(tmeda)] mapped with (*a*) *d*_norm_ and (*b*) the mol­ecular electrostatic potential.

**Figure 4:**
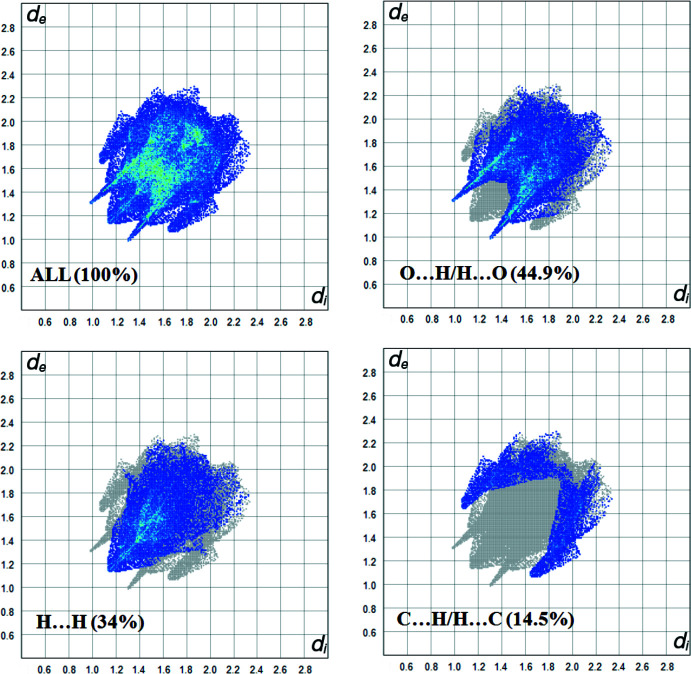
Two-dimensional fingerprint plots for [Cu(2-nitro­benzoate)_2_(tmeda)] showing all inter­actions and those delineated into O⋯H/H⋯O, H⋯H and C⋯H/H⋯C contacts (*d*_i_ is the closest inter­nal distance from a given point on the Hirshfeld surface and *d*_e_ is the closest external contact).

Hirshfeld surface analysis and the associated two-dimensional fingerprint plots (Spackman & Jayatilaka, 2009 ▸) are very important for explaining the inter­molecular contacts in the crystal structure (Demircioğlu *et al.*, 2019 ▸; Ilmi *et al.*, 2020 ▸). We performed the Hirshfeld surface analysis with *CrystalExplorer17* (Turner *et al.*, 2017 ▸). Fig. 3 ▸ shows the Hirshfeld surface mapped over *d*_norm_ (–0.2250 to 1.2935 a.u.) and the mol­ecular electrostatic potentials (–0.2173 to 0.1248). In Fig. 3 ▸ *a*, the red spots correspond to the O⋯H contacts. The electrostatic potential (Fig. 3 ▸ *b*) shows donor (red) and acceptor (blue) regions. O⋯H/H⋯O (44.9%) contacts, seen as a pair of spikes of scattered points in the fingerprint plot, make the largest contribution to the total Hirshfeld surface in [Cu(2-nitro­benzoate)_2_(tmeda)] (Fig. 4 ▸). The second most important inter­action is H⋯H, contributing 34% to the overall crystal packing, which is shown in the 2D fingerprint of the (*d*_i_, *d*_e_) points related to the H atoms. Two symmetrical wings on the left and right sides are shown in the graph of C⋯H/H⋯C inter­actions (14.5%). The Hirshfeld surface analysis confirms the importance of H-atom contacts in establishing the packing. The large number of O⋯H, H⋯H and C⋯H inter­actions suggest that van der Waals inter­actions and hydrogen bonding play the major role in the crystal packing.

## Synthesis and crystallization   

An aqueous solution of sodium 2-nitro­benzoate (5 mmol, 0.9 g) was added to an aqueous solution of CuSO_4_·5H_2_O (2.5 mmol, 0.6 g) under stirring. Tetra­methyl­ethylenedi­amine (2.5 mmol, 0.3 g) was added and the color changed from light blue to violet. The mixture was filtered and the filtrate was allowed to stand for slow evaporation. Single crystals suitable for X-ray were obtained after several days.

## Refinement   

Crystal data, data collection and structure refinement details are summarized in Table 3 ▸. C-bound H atoms were positioned geometrically (C—H = 0.95, 0.98 and 0.99 Å) and refined using a riding model, with *U*_iso_(H) = 1.5*U*_eq_(C) for methyl H atoms and 1.2*U*_eq_(C) otherwise.

## Supplementary Material

Crystal structure: contains datablock(s) I. DOI: 10.1107/S2056989021002802/zn2005sup1.cif

Structure factors: contains datablock(s) I. DOI: 10.1107/S2056989021002802/zn2005Isup2.hkl

CCDC reference: 1910225

Additional supporting information:  crystallographic information; 3D view; checkCIF report

## Figures and Tables

**Table 1:** Selected bond lengths (Å)

C1—N1	1.482 (2)	Cu1—O1	1.9589 (11)
C2—N1	1.483 (2)	Cu1—N1	2.0269 (13)
C3—N1	1.490 (2)	N2—O4	1.2206 (18)
C10—N2	1.4742 (19)	N2—O3	1.2249 (19)

**Table 2:** Hydrogen-bond geometry (Å, °)

*D*—H⋯*A*	*D*—H	H⋯*A*	*D*⋯*A*	*D*—H⋯*A*
C3—H3*A*⋯O3^i^	0.99	2.59	3.531 (2)	158
C9—H9⋯O4^ii^	0.95	2.42	3.291 (2)	152

Symmetry codes: (i) -x+1, y-1, -z+{\script{1\over 2}}; (ii) -x+{\script{3\over 2}}, -y+{\script{3\over 2}}, -z+1.

**Table 3:** Experimental details

Crystal data	
Chemical formula	[Cu(C_7_H_4_NO_4_)_2_(C_6_H_16_N_2_)]
*M*_r_	511.97
Crystal system, space group	Monoclinic, *C*2/*c*
Temperature (K)	100
*a*, *b*, *c* (Å)	12.7286 (3), 7.4918 (2), 22.8967 (6)
β (°)	98.395 (1)
*V* (Å^3^)	2160.04 (10)
*Z*	4
Radiation type	Mo *K*α
μ (mm^−1^)	1.07
Crystal size (mm)	0.22 × 0.20 × 0.12

Data collection	
Diffractometer	Bruker D8 Quest withPhoton II CPADs detector
Absorption correction	Multi-scan (*SADABS*; Bruker, 2017 ▸)
*T*_min_, *T*_max_	0.77, 0.88
No. of measured, independent and observed [*I* > 2σ(*I*)] reflections	23494, 4737, 3573
*R*_int_	0.056
(sin θ/λ)_max_ (Å^−1^)	0.808

Refinement	
*R*[*F* ^2^ > 2σ(*F* ^2^)], *wR*(*F* ^2^), *S*	0.042, 0.092, 1.03
No. of reflections	4737
No. of parameters	152
H-atom treatment	H-atom parameters constrained
Δρ_max_, Δρ_min_ (e Å^−3^)	0.55, −0.63

Computer programs: *APEX3* and *SAINT* (Bruker, 2017 ▸), *SHELXT2014/5* (Sheldrick, 2015*a* ▸) and *SHELXL2017/1* (Sheldrick, 2015*b* ▸).

## supplementary crystallographic information

### Crystal data

**Table:**

[Cu(C_7_H_4_NO_4_)_2_(C_6_H_16_N_2_)]	*F*(000) = 1060
*M**_r_* = 511.97	*D*_x_ = 1.574 Mg m^−^^3^
Monoclinic, *C*2/*c*	Mo *K*α radiation, λ = 0.71073 Å
*a* = 12.7286 (3) Å	Cell parameters from 6607 reflections
*b* = 7.4918 (2) Å	θ = 3.2–34.7°
*c* = 22.8967 (6) Å	µ = 1.07 mm^−^^1^
β = 98.395 (1)°	*T* = 100 K
*V* = 2160.04 (10) Å^3^	Block, blue
*Z* = 4	0.22 × 0.20 × 0.12 mm

### Data collection

**Table:**

Bruker D8 Quest withPhoton II CPADs detector diffractometer	4737 independent reflections
Radiation source: Incoatec microfocus source, Bruker D8 Quest	3573 reflections with *I* > 2σ(*I*)
Multilayer Mirror monochromator	*R*_int_ = 0.056
Detector resolution: 7.4074 pixels mm^-1^	θ_max_ = 35.1°, θ_min_ = 3.2°
phi and ω scans	*h* = −20→20
Absorption correction: multi-scan (SADABS; Bruker, 2017)	*k* = −12→12
*T*_min_ = 0.77, *T*_max_ = 0.88	*l* = −36→34
23494 measured reflections	

### Refinement

**Table:**

Refinement on *F*^2^	Primary atom site location: dual
Least-squares matrix: full	Hydrogen site location: inferred from neighbouring sites
*R*[*F*^2^ > 2σ(*F*^2^)] = 0.042	H-atom parameters constrained
*wR*(*F*^2^) = 0.092	*w* = 1/[σ^2^(*F*_o_^2^) + (0.0258*P*)^2^ + 3.2488*P*] where *P* = (*F*_o_^2^ + 2*F*_c_^2^)/3
*S* = 1.03	(Δ/σ)_max_ < 0.001
4737 reflections	Δρ_max_ = 0.55 e Å^−^^3^
152 parameters	Δρ_min_ = −0.63 e Å^−^^3^
0 restraints	

### Special details

**Table:**

Geometry. All esds (except the esd in the dihedral angle between two l.s. planes) are estimated using the full covariance matrix. The cell esds are taken into account individually in the estimation of esds in distances, angles and torsion angles; correlations between esds in cell parameters are only used when they are defined by crystal symmetry. An approximate (isotropic) treatment of cell esds is used for estimating esds involving l.s. planes.

### Fractional atomic coordinates and isotropic or equivalent isotropic displacement parameters (Å^2^)

**Table:**

	*x*	*y*	*z*	*U*_iso_*/*U*_eq_	
C1	0.29927 (12)	0.0971 (2)	0.26549 (9)	0.0226 (3)	
H1A	0.282285	0.110738	0.222582	0.034*	
H1B	0.282610	0.208122	0.284815	0.034*	
H1C	0.257167	−0.000768	0.278536	0.034*	
C2	0.43747 (14)	0.0376 (2)	0.34649 (8)	0.0227 (3)	
H2A	0.395284	−0.060381	0.359361	0.034*	
H2B	0.419573	0.148951	0.365245	0.034*	
H2C	0.513178	0.011891	0.357879	0.034*	
C3	0.44109 (12)	−0.1106 (2)	0.25164 (8)	0.0191 (3)	
H3A	0.400440	−0.116874	0.211384	0.023*	
H3B	0.422415	−0.215879	0.274155	0.023*	
C4	0.51601 (12)	0.4529 (2)	0.34319 (7)	0.0161 (3)	
C5	0.48616 (11)	0.56860 (19)	0.39205 (7)	0.0141 (3)	
C6	0.37979 (12)	0.5852 (2)	0.39976 (7)	0.0164 (3)	
H6	0.326473	0.529299	0.372478	0.020*	
C7	0.35056 (12)	0.6815 (2)	0.44637 (8)	0.0196 (3)	
H7	0.277599	0.693902	0.450177	0.024*	
C8	0.42749 (12)	0.7599 (2)	0.48755 (7)	0.0205 (3)	
H8	0.407256	0.823813	0.519973	0.025*	
C9	0.53414 (12)	0.7451 (2)	0.48142 (7)	0.0188 (3)	
H9	0.587555	0.797819	0.509431	0.023*	
C10	0.56064 (11)	0.6518 (2)	0.43364 (7)	0.0151 (3)	
Cu1	0.500000	0.25244 (3)	0.250000	0.01202 (6)	
N1	0.41383 (10)	0.05640 (17)	0.28142 (6)	0.0152 (2)	
N2	0.67360 (10)	0.65314 (18)	0.42590 (6)	0.0176 (3)	
O1	0.44317 (9)	0.43232 (14)	0.29904 (5)	0.0165 (2)	
O2	0.60401 (9)	0.37907 (16)	0.34882 (6)	0.0226 (2)	
O3	0.69761 (10)	0.73929 (18)	0.38435 (6)	0.0275 (3)	
O4	0.73708 (9)	0.57614 (18)	0.46249 (6)	0.0268 (3)	

### Atomic displacement parameters (Å^2^)

**Table:**

	*U*^11^	*U*^22^	*U*^33^	*U*^12^	*U*^13^	*U*^23^
C1	0.0144 (6)	0.0213 (7)	0.0324 (10)	−0.0007 (6)	0.0050 (6)	−0.0020 (7)
C2	0.0297 (8)	0.0196 (7)	0.0197 (8)	−0.0011 (6)	0.0063 (7)	0.0039 (6)
C3	0.0187 (6)	0.0126 (6)	0.0265 (8)	−0.0024 (5)	0.0054 (6)	−0.0010 (6)
C4	0.0175 (6)	0.0128 (6)	0.0184 (7)	−0.0012 (5)	0.0039 (5)	−0.0002 (5)
C5	0.0144 (6)	0.0124 (6)	0.0159 (7)	0.0005 (5)	0.0030 (5)	0.0005 (5)
C6	0.0140 (6)	0.0167 (6)	0.0187 (7)	−0.0013 (5)	0.0025 (5)	0.0003 (6)
C7	0.0161 (6)	0.0231 (7)	0.0211 (8)	0.0007 (6)	0.0073 (6)	0.0002 (6)
C8	0.0201 (6)	0.0241 (7)	0.0189 (7)	0.0001 (6)	0.0073 (6)	−0.0038 (7)
C9	0.0178 (6)	0.0204 (6)	0.0181 (7)	−0.0009 (6)	0.0026 (5)	−0.0021 (6)
C10	0.0131 (6)	0.0149 (6)	0.0177 (7)	0.0004 (5)	0.0036 (5)	−0.0002 (5)
Cu1	0.01247 (10)	0.01009 (10)	0.01356 (12)	0.000	0.00210 (8)	0.000
N1	0.0149 (5)	0.0127 (5)	0.0186 (6)	−0.0002 (4)	0.0040 (5)	0.0006 (5)
N2	0.0145 (5)	0.0174 (6)	0.0214 (7)	−0.0017 (5)	0.0037 (5)	−0.0035 (5)
O1	0.0189 (5)	0.0143 (5)	0.0161 (5)	0.0004 (4)	0.0021 (4)	−0.0023 (4)
O2	0.0180 (5)	0.0237 (6)	0.0262 (6)	0.0038 (4)	0.0038 (5)	−0.0065 (5)
O3	0.0213 (5)	0.0332 (7)	0.0297 (7)	−0.0039 (5)	0.0092 (5)	0.0044 (6)
O4	0.0165 (5)	0.0295 (7)	0.0329 (7)	0.0040 (5)	−0.0013 (5)	0.0026 (6)

### Geometric parameters (Å, º)

**Table:**

C1—N1	1.482 (2)	C5—C6	1.396 (2)
C1—H1A	0.9800	C6—C7	1.383 (2)
C1—H1B	0.9800	C6—H6	0.9500
C1—H1C	0.9800	C7—C8	1.387 (2)
C2—N1	1.483 (2)	C7—H7	0.9500
C2—H2A	0.9800	C8—C9	1.389 (2)
C2—H2B	0.9800	C8—H8	0.9500
C2—H2C	0.9800	C9—C10	1.381 (2)
C3—N1	1.490 (2)	C9—H9	0.9500
C3—C3^i^	1.513 (3)	C10—N2	1.4742 (19)
C3—H3A	0.9900	Cu1—O1	1.9589 (11)
C3—H3B	0.9900	Cu1—O1^i^	1.9589 (11)
C4—O2	1.2388 (18)	Cu1—N1	2.0269 (13)
C4—O1	1.2772 (19)	Cu1—N1^i^	2.0269 (13)
C4—C5	1.508 (2)	N2—O4	1.2206 (18)
C5—C10	1.389 (2)	N2—O3	1.2249 (19)
			
N1—C1—H1A	109.5	C6—C7—H7	119.9
N1—C1—H1B	109.5	C8—C7—H7	119.9
H1A—C1—H1B	109.5	C7—C8—C9	120.04 (15)
N1—C1—H1C	109.5	C7—C8—H8	120.0
H1A—C1—H1C	109.5	C9—C8—H8	120.0
H1B—C1—H1C	109.5	C10—C9—C8	118.42 (14)
N1—C2—H2A	109.5	C10—C9—H9	120.8
N1—C2—H2B	109.5	C8—C9—H9	120.8
H2A—C2—H2B	109.5	C9—C10—C5	123.28 (14)
N1—C2—H2C	109.5	C9—C10—N2	116.55 (13)
H2A—C2—H2C	109.5	C5—C10—N2	120.05 (13)
H2B—C2—H2C	109.5	O1—Cu1—O1^i^	93.07 (7)
N1—C3—C3^i^	108.76 (11)	O1—Cu1—N1	91.76 (5)
N1—C3—H3A	109.9	O1^i^—Cu1—N1	165.06 (5)
C3^i^—C3—H3A	109.9	O1—Cu1—N1^i^	165.06 (5)
N1—C3—H3B	109.9	O1^i^—Cu1—N1^i^	91.76 (5)
C3^i^—C3—H3B	109.9	N1—Cu1—N1^i^	87.13 (7)
H3A—C3—H3B	108.3	C1—N1—C2	108.35 (13)
O2—C4—O1	124.71 (15)	C1—N1—C3	110.27 (12)
O2—C4—C5	120.10 (14)	C2—N1—C3	110.70 (13)
O1—C4—C5	115.09 (13)	C1—N1—Cu1	109.12 (10)
C10—C5—C6	116.79 (14)	C2—N1—Cu1	112.61 (10)
C10—C5—C4	123.09 (13)	C3—N1—Cu1	105.77 (9)
C6—C5—C4	119.95 (13)	O4—N2—O3	124.54 (14)
C7—C6—C5	121.26 (14)	O4—N2—C10	118.28 (14)
C7—C6—H6	119.4	O3—N2—C10	117.09 (13)
C5—C6—H6	119.4	C4—O1—Cu1	104.50 (9)
C6—C7—C8	120.18 (14)		
			
O2—C4—C5—C10	24.2 (2)	C4—C5—C10—C9	−174.19 (15)
O1—C4—C5—C10	−159.09 (14)	C6—C5—C10—N2	−174.93 (13)
O2—C4—C5—C6	−150.89 (15)	C4—C5—C10—N2	9.9 (2)
O1—C4—C5—C6	25.8 (2)	C3^i^—C3—N1—C1	157.97 (16)
C10—C5—C6—C7	0.6 (2)	C3^i^—C3—N1—C2	−82.14 (18)
C4—C5—C6—C7	175.98 (15)	C3^i^—C3—N1—Cu1	40.11 (18)
C5—C6—C7—C8	−1.8 (2)	C9—C10—N2—O4	67.41 (19)
C6—C7—C8—C9	1.3 (3)	C5—C10—N2—O4	−116.36 (17)
C7—C8—C9—C10	0.2 (2)	C9—C10—N2—O3	−109.26 (17)
C8—C9—C10—C5	−1.5 (2)	C5—C10—N2—O3	66.97 (19)
C8—C9—C10—N2	174.64 (15)	O2—C4—O1—Cu1	4.07 (19)
C6—C5—C10—C9	1.0 (2)	C5—C4—O1—Cu1	−172.47 (10)

Symmetry code: (i) −*x*+1, *y*, −*z*+1/2.

### Hydrogen-bond geometry (Å, º)

**Table:**

*D*—H···*A*	*D*—H	H···*A*	*D*···*A*	*D*—H···*A*
C3—H3*A*···O3^ii^	0.99	2.59	3.531 (2)	158
C9—H9···O4^iii^	0.95	2.42	3.291 (2)	152

Symmetry codes: (ii) −*x*+1, *y*−1, −*z*+1/2; (iii) −*x*+3/2, −*y*+3/2, −*z*+1.
